# Reproductive ecology and isolation of *Psittacanthus calyculatus* and *P. auriculatus* mistletoes (Loranthaceae)

**DOI:** 10.7717/peerj.2491

**Published:** 2016-09-27

**Authors:** Sergio Díaz Infante, Carlos Lara, María del Coro Arizmendi, Luis E. Eguiarte, Juan Francisco Ornelas

**Affiliations:** 1 Laboratorio de Ecología, UBIPRO, Facultad de Estudios Superiores Iztacala, Universidad Nacional Autónoma de México, Tlalnepantla de Baz, Estado de México, Mexico; 2 Centro de Investigación en Ciencias Biológicas, Universidad Autónoma de Tlaxcala, Tlaxcala, Mexico; 3 Departamento de Ecología Evolutiva, Instituto de Ecología, Universidad Nacional Autónoma de México, Ciudad de México, Distrito Federal, Mexico; 4 Departamento de Biología Evolutiva, Instituto de Ecología, A.C., Xalapa, Veracruz, Mexico

**Keywords:** Allopatry, Bird pollination, Character displacement, Hemiparasites, Hummingbird, Mexico, Loranthaceae

## Abstract

**Background:**

Relationships between floral biology and pollinator behavior are important to understanding species diversity of hemiparasitic *Psittacanthus* mistletoes (c. 120 species). We aimed to investigate trait divergence linked to pollinator attraction and reproductive isolation (*RI*) in two hummingbird-pollinated and bird-dispersed *Psittacanthus* species with range overlap.

**Methods:**

We investigated the phylogenetic relationships, floral biology, pollinator assemblages, seed dispersers and host usage, and the breeding system and female reproductive success of two sympatric populations of *P. calyculatus* and *P. auriculatus*, and one allopatric population of *P. calyculatus*. Flowers in sympatry were also reciprocally pollinated to assess a post-mating component of *RI*.

**Results:**

Hummingbird assemblages differed between *calyculatus* populations, while allopatric plants of *calyculatus* opened more but smaller flowers with longer lifespans and produced less nectar than those in sympatry. Bayesian-based phylogenetic analysis indicated monophyly for *calyculatus* populations (i.e. both populations belong to the same species). In sympatry, *calyculatus* plants opened more and larger flowers with longer lifespans and produced same nectar volume than those of *auriculatus*; populations shared pollinators but seed dispersers and host usage differed between species. Nectar standing crops differed between sympatric populations, with lower visitation in *calyculatus*. Hand pollination experiments indicated a predominant outcrossing breeding system, with fruit set after interspecific pollination two times higher from *calyculatus* to *auriculatus* than in the opposite direction.

**Conclusions:**

Given the low genetic differentiation between *calyculatus* populations, observed trait divergence could have resulted from changes regarding the local communities of pollinators and, therefore, expected divergence for peripheral, allopatric populations. Using *RI* estimates, there were fewer heterospecific matings than expected by chance in *P. calyculatus* (*RI_4A_* = 0.629) as compared to *P. auriculatus* (*RI_4A_* = 0.20). When considering other factors of ecological isolation that affect co-occurrence, the *RI_4C_* values indicate that isolation by hummingbird pollinators was less effective (0.20) than isolation by host tree species and seed dispersers (0.80 and 0.60, respectively), suggesting that host usage is the most important ecological isolation factor between the two species. Accordingly, the absolute and relative cumulative strength values indicated that the host tree species’ barrier is currently contributing the most to maintaining these species in sympatry.

## Introduction

Floral divergence mediated by pollinator selection pressures is considered to be a primary driver of plants speciation (Coyne & Orr, 2004). Simple shifts in flower color, scent and form have all been implicated in the evolution of strong reproductive isolation (*RI*) between species, in which gene flow is reduced due to a pre- or post-pollination barrier for interspecific crosses compared with intraspecific crosses (reviewed in Widmer, Lexer & Cozzolino, 2009; Briscoe Runquist & Moeller, 2014; Baack et al., 2015). These traits confer *RI* primarily because they result in the differential attraction or deterrence of (suites of) pollinators. However, all components of *RI* have been measured in a small albeit growing number of taxa, with ecogeographic barriers estimated for few cases (Baack et al., 2015). The mechanisms of *RI* in plant speciation include divergent selection across habitats favoring phenological, pollinator or mating system shifts (e.g., Grossenbacher & Whittall, 2011). During the process of speciation and beyond, mating system transitions (i.e. from outcrossing to selfing) can dramatically influence the potential for gene exchange, competition for pollinators, and ecological differentiation (reviewed in Baack et al., 2015; Grossenbacher et al., 2016). These transitions facilitate the evolution of *RI* between recently diverged species because of interactions between pollinator-sharing taxa where their geographical ranges overlap reducing pollen transfer between taxa (Briscoe Runquist & Moeller, 2014 and references therein).

Population differentiation is generally constrained by gene flow, thereby preventing the evolution of strong *RI* (e.g., Coyne & Orr, 2004). Because recently diverged sister species often grow in the same or similar habitat, flowering phenologies overlap, and many times share pollinators, ample opportunity for gene exchange between species exists if *RI* mechanisms are weak or lacking, and other mechanisms are expected to prevent strong hybridization and introgression in the absence of high pollinator specificity (Rieseberg & Willis, 2007; Lowry et al., 2008; Johnson et al., 2015). Although pre-pollination barriers are often very strong and contribute more to total *RI* than post-pollination barriers (Lowry et al., 2008; Baack et al., 2015), in some cases the potential presence of post-zygotic barriers (i.e. reduced fitness of hybrids) may be strong as well during the process of speciation.

Mistletoes are ecologically important hemiparasites in temperate and tropical forest ecosystems as they provide food, cover and nesting sites for a variety of birds, mammals and insects (Watson, 2001). Most of these mistletoes that depend on avian vectors for pollen and seed dispersal infect a large number of tree species including commercially important coniferous and other hardwood timber stands (Mathiasen et al., 2008). *Psittacanthus* mistletoes (Loranthaceae, ∼120 species; Kuijt, 2009; Kuijt, 2014) are aerial hemiparasites distributed throughout the Neotropics on a wide range of host tree species, from central Baja California and Sonora south through Mesoamerica to Bolivia and northern Argentina (Kuijt, 2009). There is virtually no distributional overlap between Mesoamerican and South American species in the genus. Within Mesoamerica, however, several species overlap extensively (Kuijt, 2009). Closely related species, often in sympatry, usually parasitize distantly related host species suggesting strong host local adaptation, whereas *Psittacanthus* species that infect closely related host species have allopatric distributions (Kuijt, 2009; Ornelas et al., 2016). The tendency for closely related mistletoes to infect distantly related host species supports the host-switching model of mistletoe speciation (Norton & Carpenter, 1998), in which mistletoe speciation in sympatry would occur by changing host specificity leading to specialization on different hosts and the formation of mistletoe races. It would then appear that the same evolutionary forces in the origin of ecogeographic barriers are at work within species (i.e., mistletoe races).

Although *Psittacanthus* is the most diverse genus of the family in the Neotropics (Kuijt, 2009), knowledge on floral biology and breeding systems is limited to single populations of few species (Azpeitia & Lara, 2006; Leal, Lopes & Machado, 2006; Ramírez & Ornelas, 2010; Arruda et al., 2012; Guerra, Galetto & Silva, 2014; Pérez-Crespo et al., 2016). *Psittacanthus* species possess flowers with a great variety of colors, sizes and shapes, but typically species of this genus possess large, brilliant and tubular flowers with inflorescences forming triads or sometimes dyads (Kuijt, 2009; Arruda et al., 2012). This variety of flowers attracts a diverse array of floral visitors, including butterflies, bumble bees, hummingbirds, several species of parrots and passerine birds, and nectar-feeding bats (Ramírez & Ornelas, 2010; Arruda et al., 2012; Guerra, Galetto & Silva, 2014; Pérez-Crespo et al., 2016).

Sympatric populations of closely related *Psittacanthus* species with flowering overlap and potential for interspecific pollen flow, represent a good system to investigate the contribution of pollinators to *RI*, the maintenance of species boundaries and interspecific hybridization. When two species overlap geographically, the differences between them are accentuated in the area of sympatry and weakened or lost entirely as they become allopatric (‘character displacement;’ e.g., Brown & Wilson, 1956; Schluter & McPhail, 1992; Briscoe Runquist & Moeller, 2014). Character displacement would strength pre-pollination reproductive barriers between sympatric species by encouraging the divergence of floral traits linked to outcrossed reproduction driven by competition for effective pollinators and, as a consequence, a pollinator shift might occur (Hopkins, 2013). Studies of character displacement usually involve comparisons of traits in question between allopatric and sympatric populations of closely related species. When the traits in question are more divergent in areas of sympatry than in areas of allopatry, the divergence is often explained as arising from selection against costly interspecific mating in the case of reproductive character displacement or from selection against interspecific resource competition in the case of ecological character displacement (Jang & Gerhardt, 2006). Nonetheless, the evolution of *RI*, caused by selection against maladapted hybrids or costly mating between closely related diverging taxa (‘reinforcement’), does not always result in a pattern of divergence (reviewed in Hopkins (2013)).

Our work focuses on the pollination biology and breeding system of *Psittacanthus calyculatus* (DC.) G. Don in partial overlap with *P. auriculatus* (Oliv.) Eichler, and compared that with data from an allopatric *P. calyculatus* population, to investigate trait divergence and *RI* and related reinforcement processes. Pollination biology was examined by describing the floral longevity and morphology, nectar production dynamics and floral visitors of *P. calyculatus* in sympatry with *P. auriculatus* and compared that with allopatric *P. calyculatus*, and the breeding system through intraspecific and interspecific hand pollination experiments with reproductive success estimated as number of fruits produced over the number of flowers naturally or hand pollinated. The two species are closely related (Ornelas et al., 2016) and the mechanisms that restrict gene flow between these potentially interbreeding populations need to be elucidated. For sympatric closely related plant species, factors contributing to *RI* may involve pre-pollination differences in flowering phenology, pollinator fidelity and/or variations in mating systems (Pascarella, 2007; Ramsey, Bradshaw & Schemske, 2003; Brys et al., 2014; Grossenbacher et al., 2016), while post-pollination isolation may involve gamete incompatibility and/or pollen tube competition (Coyne & Orr, 2004).

The study was centered on the following questions: (1) what are the phylogenetic relationships between *P. auriculatus* and *P. calyculatus*; (2) Is the sympatric population of *P. calyculatus* genetically differentiated of other allopatric *P. calyculatus* populations; (3) What is the contribution of reproductive barriers to pollination success of intraspecific and interspecific pollination between sympatric populations of *P. calyculatus* and *P. auriculatus*; and (4) the significance of ecological isolating factors (e.g., pollinator, host and seed disperser sharing) in their *RI*? Our study represents a first view of the breeding system and pollination ecology of these two *Psittacanthus* species in sympatry. Using *RI* measures (i.e., deviations from random matings; Sobel & Chen, 2014), we aim to determine the relative strength among pre-pollination barriers in their *RI*.

## Methods

### Field study permissions

We obtained collecting permits from the Mexican government to conduct this work from the Secretaría de Medio Ambiente y Recursos Naturales, Instituto Nacional de Ecología, Dirección General de Vida Silvestre (permit number: (INE, SEMARNAT, SGPA/DGGFS/712/1299/12). The collecting permit specifically allowed for the collection of leaf tissue samples described in this study as newly sequenced. We had access to the material under the terms of the scientific permit and no specific permits for fieldwork were required to work at the study areas though municipal and community authorities of Nativitas and Tetlatlahuca, Tlaxcala, and Santiago Matatlán, Oaxaca were informed. Leaf tissue samples were obtained from the plant species reported here with no further manipulation.

### Study species

*Psittacanthus calyculatus* is widely distributed in Mexico, from Nayarit and Aguascalientes to Chiapas states, found at mid-elevations (1,000–2,600 meters above sea level, m a.s.l.; Calderón de Rzedowski & Rzedowski, 2002). Its flowers are isomorphic, straight or slightly inclined distally, and yellowish orange to bright scarlet or somewhat pinkish (Kuijt, 2009). The species is extremely variable in flower morphology, being sometimes difficult to separate from other *Psittacanthus* species (Kuijt, 2009). It grows on various host trees in central Mexico including species of *Quercus*, *Acacia* and *Prosopis* in undisturbed areas and *Crataegus*, *Salix* and *Prunus* in suburban and agricultural landscapes (Azpeitia & Lara, 2006; Kuijt, 2009; Lara, Pérez & Ornelas, 2009; Arce, 2012; Zuria, Castellanos & Gates, 2014). In suburban Tlaxcala (3 km from Tlaxcala City at 2,200 m a.s.l.), *P. calyculatus* parasitizes several host tree species such as *Alnus acuminata*, *Salix babylonica* and *Populus* spp., as well as several tree species of local economic interest such as *Crataegus mexicana*, *Persea americana*, *Prunus serotina* and *Malus domestica* (Acosta, Cházaro & Patiño, 1993; Azpeitia & Lara, 2006; Lara, Pérez & Ornelas, 2009). Flowers last an average of 6 days in this population, with anther dehiscence occurring during the first 24 h prior stigma receptivity, stigmas being more receptive during the third day. Flowering coincides with the summer rainy season from ending June to September (Azpeitia & Lara, 2006). Hummingbirds are the main floral visitors of *P. calyculatus* (Azpeitia & Lara, 2006) and several bird species consume and disperse its one-seeded fleshy fruits (Zuria, Castellanos & Gates, 2014).

**Figure 1:**
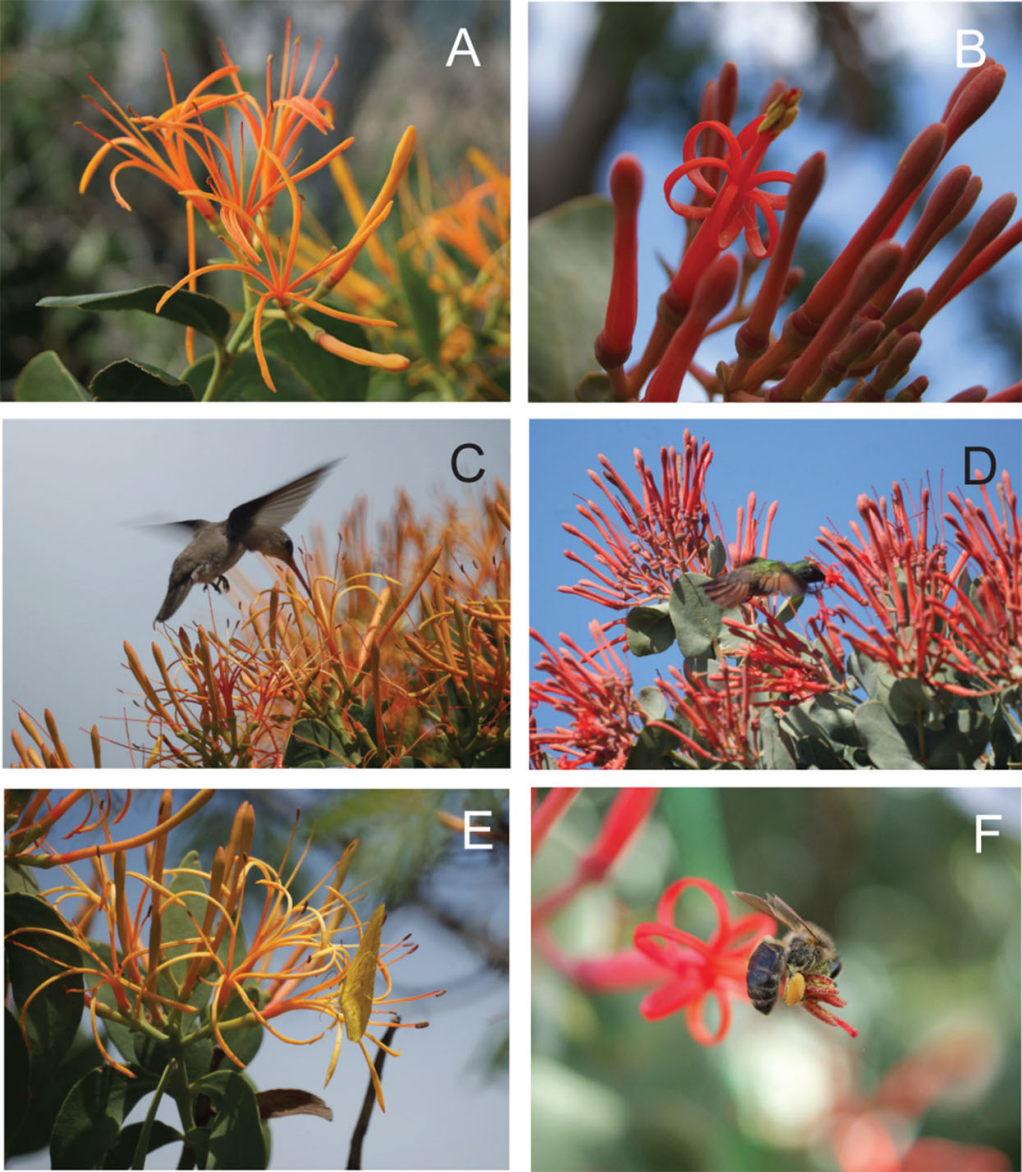
Morphology and floral visitors of *Psittacanthus calyculatus* and *P. auriculatus* in an Acacia-grassland at Santiago Matatlán, Oaxaca, Mexico. (A) Inflorescence and flower morphology of *P. calyculatus*. Note that the yellow to bright orange flowers do not form a discernable floral tube during anthesis, petals strongly curl around, filaments are extremely long, and that stamens are spreading out. (B) Inflorescence and flower morphology of *P. auriculatus*. Note the open flower with recently dehiscent anthers and yellow pollen (staminate). Flowers are bright red-pink color, petals form a floral tube, which remains tubular with age, and filaments and stamens tightly clustered remain erected during anthesis favoring pollination by hummingbirds while removing nectar from the top. (C) *Amazilia violiceps* hovering and taking nectar during a visit to *P. calyculatus*. Note that when hovering hummingbirds visit a *P. calyculatus* flower the anthers and stigma make contact with several areas of their body. (D) *Amazilia beryllina* hovering on the flower of *P. auriculatus* while accessing nectar. Note that when hovering hummingbirds visit a *P. auriculatus* flower the anthers and stigma make contact with their foreheads. (E) *Anteos clorinde* resting on a *P. calyculatus* flower, while accessing nectar. Note that when butterflies perch on a *P. calyculatus* flower while accessing nectar, the anthers and stigma make no contact with their bodies. (F) One-day staminate flower of *P. auriculatus* with empty anthers after pollen collection by honeybees *Apis mellifera* (see also Pérez-Crespo et al., 2016). Photos by María José Pérez-Crespo.

*Psittacanthus auriculatus* is the only Mesoamerican species with cordate leaves (Kuijt, 2009). It is found mainly on *Acacia* trees at mid-elevations (1,300–2,000 m a.s.l.). The flowers of *P. auriculatus* are nearly isomorphic, straight and stout, red to orange (Kuijt, 2009), partially protandrous and hummingbird pollinated (Pérez-Crespo et al., 2016). The species differ in many morphological traits, especially those related to corolla characteristics (Fig. 1). The two species in general have ranges that do not overlap, however, *P. auriculatus* co-occurs with *P. calyculatus* in some localities of the states of Puebla and Oaxaca to which *P. auriculatus* is restricted (Kuijt, 2009). Under these sharing habitat circumstances, other ecological factors such as the composition of pollinator assemblages and seed dispersers and host usage, may maintain boundaries between closely related plant species in the absence of pollinator specificity (Rieseberg & Willis, 2007; Lowry et al., 2008; Johnson et al., 2015).

### Study sites

*Psittacanthus calyculatus* and *P. auriculatus* mistletoes were studied in sympatry (*P. calyculatus* and *P. auriculatus* in Oaxaca; hereafter CO and AO, respectively) from August 2013 to January 2014 on bordering tree lines of agricultural areas in Santiago Matatlán, Oaxaca, Mexico (16°50′53″N, 96°22′18″W; at 1,784 m a.s.l.; Table S1). The study area is a dry region characterized by a summer rainy season (May–October) and mean annual precipitation of 635 mm, with a minimum of 208 mm in December and a maximum of 1,198 mm in July. The mean annual temperature is 20 °C, with a range of 8–12 °C minimum in December and January and a maximum range of 28–35 °C from March to May (http://www.inafed.gob.mx/work/enciclopedia/EMM20oaxaca/index.html). Infected trees in the study area include *Acacia* spp., *Prosopis laevigata*, *Celtis caudata*, *Bursera* spp., *Eysenhartia polystachya* and *Byrsonima crassifolia* (S. Díaz Infante, 2015, unpublished data). The infection prevalence (the percentage of infected host trees per species) in the area is 7–15%, with *Celtis caudata* trees most affected by *P. calyculatus* (57%) and *Acacia schaffneri* (86%) by *P. auriculatus* (S. Díaz Infante, 2015, unpublished data). Although one *Acacia* individual was infected by both *Psittacanthus* species, natural hybrids have not been found at the area of sympatry.

Fieldwork of allopatric *P. calyculatus* mistletoes (*P. calyculatus* in Tlaxcala; hereafter CT) was done from July 2013 to January 2014 on individuals growing on host trees bordering agricultural areas and remnants of oak forest in the surroundings of Hacienda Santa Agueda, located in the W slope of the La Malinche volcano between the municipalities of Nativitas and Tetlatlahuca, Tlaxcala, Mexico (19°11′24″N, 98°17′14″W; at 2,200 m a.s.l.; Table S1). Climate is cold and dry throughout most of the year, with a rainy season from June to September. Mean annual precipitation is 762 mm, with a minimum of 6.3–4.4 mm in February and a maximum of 132.1–165 mm in June. The mean annual temperature is 16 °C, with a range of 0.5–7.2 °C minimum in December and January and a maximum range of 26.2–24.3 °C from March to May (http://www.inafed.gob.mx/work/enciclopedia/EMM29tlaxcala/index.html). Infected trees in the region include *Salix bonplandiana*, *Quercus* spp., *Prunus serotina*, *Populus deltoides*, *Populus alba*, *Eucaliptus camaldulensis*, *Alnus acuminata, Crataegus mexicana, Mimosa* sp., *Eysenhardtia polystachya, Fraxinus uhdei, Pyrus communis* and *Grevillea robusta* (Lara, Pérez & Ornelas, 2009; S. Díaz Infante, 2015, unpublished data). The prevalence of *P. calyculatus* infection is on average 25% of available host trees in the study area, with *Quercus* (43%) and *Salix* (52%) trees contributing most (S. Díaz Infante, 2015, unpublished data).

### Phylogenetic relationships

To understand the phylogenetic relationships between the studied populations, thirty-five individuals of *P. calyculatus* (CO, *n* = 19) and *P. auriculatus* (AO, *n* = 16) were sampled from the Oaxaca sympatric populations and 10 individuals of allopatric *P. calyculatus* (CT) from Santa Agueda, Tlaxcala. Further sets of 8–10 individuals of *P. calyculatus* from two localities in Tlaxcala (Tlaxcala, San Luis Teolocholco) and 26 individuals from two localities in Jalisco (San José de Gracia, Gómez Farías), and 12 individuals from adjacent populations near Santiago Comaltepec town, Oaxaca of closely related *P. schiedeanus*. We also collected specimens on the Yucatan Peninsula of the congeneric species *P. mayanus* (*n* = 23) for phylogenetic analyses to be used as outgroup, according to Ornelas et al. (2016). Locality information of the *Psittacanthus* populations used in the study is given in Table S1.

Leaf tissue samples were preserved in silica gel desiccant until DNA extractions were performed. Total genomic DNA was extracted from silica-dried material using a modified 2 × cetyl trimethyl ammonium bromide (CTAB) protocol (Doyle & Doyle, 1987) or the DNeasy Plant Mini kit (Qiagen, Valencia, CA, USA) using the manufacturer protocol.

Amplification of the nuclear nrDNA *ITS* region was conducted with the primers ITS5HP (Suh et al., 1993) and ITS4 (White et al., 1990), whereas for chloroplast *trnL-F* intergenic spacer region we used the universal primers ‘e’ and ‘f’ (Taberlet et al., 1991). For targeting successful sequencing of *ITS* region, we used the primers ITS-F2-Psitta (5′-TCGCAGTATGCTCCGTATTG-3′) and ITS-R2-Psitta (5′-TCGTAACAAGGTTTCCGTAGG-3′) designed for this project (Ornelas et al., 2016). Protocols for PCR reactions and for sequencing the PCR products are described elsewhere (Ornelas et al., 2016). PCR products were purified with the QIAquick kit (Qiagen) and sequenced in both directions to check the validity of the sequence data using the BigDye Terminator Cycle Sequencing kit (Applied Biosystems, Foster City, CA, USA). The products were analyzed on a 310 automated DNA sequencer (Applied Biosystems) at the Instituto de Ecología, AC sequencing facility, or at University of Washington High Throughput Genomics Unit, Seattle, Washington.

DNA sequences were assembled using SEQUENCHER v. 4.9 (Gene Codes, Ann Arbor, MI, USA) and then were manually aligned with SE-AL v. 2.0a111 (http://tree.bio.ed.ac.uk/software/seal). All sequences of *P. calyculatus* and *P. auriculatus* and those new of other *Psittacanthus* species have been submitted to GenBank (Accession nos. *ITS*: KX241619–KX241734, *trnL-F*: KX241735–KX241823). The sequence alignment input files available from the Dryad Digital Repository: http://dx.doi.org/10.5061/dryad.6kt60. Voucher information of the *Psittacanthus* populations used in the study is given in Table S1.

To estimate the relationships among groups of populations, we used *ITS* and *trnL-F* sequences for *P. calyculatus* samples and *BEAST (Heled & Drummond, 2010) with the multispecies coalescent model implemented in BEAST v. 1.8.0 (Drummond & Rambaut, 2007). *BEAST models the lineage sorting process between units for groups of individuals not connected by gene flow above, at, or below the species level (Heled & Drummond, 2010). *Psittacanthus mayanus* samples were used as outgroups. Nucleotide substitution models, for *ITS* and for *trnL-F* selected with jMODELTEST v. 0.1.1 (Posada, 2008) were incorporated as the Hasegawa-Kishino-Yano (HKY) model for both markers.

The simulation was first run with all samples of *P. calyculatus* as one lineage (CALY; one-species hypothesis), then with samples as two lineages corresponding to samples of the population from Santiago Matatlán, Oaxaca, Mexico (in sympatry with *P. auriculatus*; two-species hypothesis) and samples from the other *P. calyculatus* populations or three separate lineages according to geography (Oaxaca, Tlaxcala, Jalisco; three-species hypothesis). We ran *BEAST two times for 30 million generations, sampling every 1,000 steps, using a Yule speciation tree prior, relaxed clock model with an uncorrelated lognormal distribution, and the mean mutation rates of 4.13 × 10^−9^ s/s/y for *ITS* of herbaceous annual/perennial plants (Kay, Whittall & Hodges, 2006) and 8.24 × 10^−9^ s/s/y for the *trnL-F* estimated for annual or perennial herbs (Richardson et al., 2001). After the analysis in BEAST, log and tree files were combined using LOGCOMBINER v. 1.8.0 (Drummond & Rambaut, 2007) and summarized as a maximum clade credibility tree using TREEANNOTATOR v. 1.8.0 (Drummond & Rambaut, 2007) with a burn-in of 25%. We used the software TRACER v. 1.6 (http://tree.bio.ed.ac.uk/software/tracer/) to visualize the results of the runs and to check the ESS (cut off values > 50) of each parameter. The likelihood scores under the species delimitation hypothesis (see Results) were compared with ln Bayes factors (BF) tests to determine which species assignment significantly improved explanation of the data.

### Floral and fruit display

We haphazardly selected and tagged 15 mistletoes (one from each of 15 host trees) from each of two populations of *Psittacanthus calyculatus* and *P. auriculatu*s in sympatry (CO and AO, respectively) and from the *P. calyculatus* population in allopatry (CT) to record total floral display (number of buds and number of open flowers per plant) in August 2013 and total fruit display (number of fruits/plant) on the same individuals in October.

### Flower longevity

To determine flower longevity, we haphazardly chose and marked a total of 30 floral buds about to open from 10 mistletoe plants (three floral buds per plant) growing on 10 infected trees, and daily followed them until wilting. The same procedure was applied to individuals from the three populations (CO, AO, CT).

### Flower and fruit morphology

Flower and fruit measurements were taken on individuals from the three populations as described above. At the beginning of the flowering season (June–July 2013), we collected and measured a total of 30 flowers from inflorescences (three flowers per plant) of 10 mistletoe individuals of each species growing on 10 host trees at each site. Anther, stamen filament, style, and width of ovaries were measured with a digital caliper (error: 0.01 mm). Fruit size measurements were taken in October on 53 fruits from infructescences of 18 mistletoe individuals (three fruits per plant) growing on different host trees at each site. Fruit pedicel length, length and width of fruit were measured with a digital caliper (error: 0.01 mm).

### Nectar standing crop

Nectar standing crop data were collected in July and August 2013 from a total of 150 flowers (15 flowers per hour) from 10 mistletoe plants growing onto 10 host trees. The same measurements were conducted on flowers from the three populations (*n* = 450 flowers). We extracted each hour (from 08:00 to 17:00 h) the nectar available in 15 different flowers that had been exposed to floral visitors, and measured its volume and concentration to evaluate variation in nectar standing crops during the period of hummingbird activity. Nectar volume per flower was removed and measured by using calibrated micropipettes (5 μL) and a digital caliper. Sugar concentration (percentage sucrose) was measured with a hand-held pocket refractometer (SperScientific 300001, Scottsdale, AZ, USA; range concentration 0–32° BRIX scale), and the amount of sugar produced was expressed as milligrams of sugar after Kearns & Inouye (1993).

### Nectar secretion pattern

In a different group of *P. calyculatus* plants (10 mistletoe plants) at both sites, two buds ready to open of selected inflorescences per plant were bagged with bridal netting and excluded from floral visitors to let nectar accumulate. The accumulated nectar was extracted the following day after 24 h of the exclusion. In two additional buds ready to open per plant, nectar was removed and measured three times the following day (1st day of anthesis) at 2-h intervals (09:00, 11:00 and 13:00) to explore the capacity of *P. calyculatus* flowers to replenish the repeatedly removed nectar. Flowers remained bagged between nectar removals. Nectar volume per flower was measured as described above. Again, the same measurements were conducted on *P. auriculatus* flowers in sympatry with *P. calyculatus* for comparison.

### Floral pollinators and fruit consumers

Preliminary observations at our study sites indicated that mistletoe flowers of both *P. calyculatus* and *P. auriculatus* were mainly visited by hummingbirds. On July 2013, we haphazardly selected three host trees at each site to determine the foraging patterns of the visiting hummingbird species. Each tree was observed using binoculars from 08:00 h in the morning until noon and from 12:00 to 16:00 h (peak of visitation) during two consecutive days recording the hummingbird species and their number of visits to each of the focal mistletoe plants. Observations were made from about 10 m away from the focal mistletoe. Bees, wasps, bumblebees and butterflies were also observed foraging on mistletoe flowers and they may contribute to pollination. However, their visits were not quantified in this study because most of times we were not able to observe them to contact anthers or stigmas during their visits.

On January 2014, the same procedure was followed to determine the visiting bird species consuming mistletoe fruits.

### Breeding system

The breeding system was determined with controlled pollination experiments. We tested the responses of *P. calyculatus* to self and outcross pollen in the flowering season (July) of 2013 at both sites. One hundred and twenty flowers from 10 selected mistletoe individuals (12 flowers per plant) were tagged, emasculated prior to pollination and bagged with 1-mm bridal tulle mesh bags to exclude floral visitors.

Three pollination treatments (autogamous hand-self, hand-geitonogamy and hand-outcross) were applied to each plant species (30 flowers per treatment; three flowers per plant). This approach provided within plant controls, but treatments may compete with each other for seed resources. In the autogamous hand-self treatment, we brushed anthers of the same flower onto the stigma to pollinate flowers and excluded floral visitors by enclosing the inflorescence with mesh bags until fruit maturation. To test for self-compatibility in the hand-geitonogamy treatment, we hand-pollinated previously bagged flowers by brushing anthers from other flower of the same individual plant. In the hand-outcross treatment, flowers were tested for cross-compatibility by smearing one anther from an arbitrarily selected pollen donor onto the receptive virgin stigma of a previously bagged flower. Pollination treatments were applied once on 3-D flowers when stigma receptivity is higher (Azpeitia & Lara, 2006; see also Results).

We also assessed natural pollination and fruit set on 30 flowers (three flowers per plant) that remained unbagged and open to natural pollination. This treatment acted as a control for seed production, although the mechanisms affecting seed production in this treatment are unclear and may be due either to resource allocation or pollen load, or both, as shown in other Loranthaceae species (Robertson et al., 1999; Montgomery et al., 2003). Fruits from experimental flowers were collected and quantified per pollination treatment two months later. For comparison, the same procedures were applied to *P. auriculatus* in sympatry with *P. calyculatus*. Flowers of both species mature into purplish-black fleshy fruits containing one seed (Azpeitia & Lara, 2006; Pérez-Crespo et al., 2016).

### Reproductive isolation

To test for potential of hybridization in sympatric populations of *P. calyculatus* and *P. auriculatus*, we added a second pollination experiment and performed reciprocal crosses between each species. Interspecific pollinations were done by smearing one anther from an arbitrarily selected pollen donor of *P. calyculatus* onto the receptive virgin stigma of *P. auriculatus*, and vice versa (*n* = 30 flowers per species, three flowers per plant), in flowers emasculated prior to pollination and previously bagged to exclude pollinators. Fruits from experimental flowers were collected and quantified per species two months later.

We quantified the contributions of post-mating reproductive barriers between *P. calyculatus* and *P. auriculatus* using Sobel & Chen’s (2014) methodology for *RI* (see Supplemental Information for detailed explanations of *RI* calculations). We compared *RI* values between sympatric populations following the linear formulation suggested by Sobel & Chen (2014): *R1_4A_* = 1 − 2 * (*H*/*H* + *C*), in which *H* and *C* are heterospecific and conspecific matings (i.e., pollination success of intraspecific and interspecific pollination).

In addition, we assessed the significance of additional pre-pollination ecological isolating factors (pollinators, host tree species and seed dispersers) between sympatric populations using the methods outlined in Sobel & Chen (2014) using the equation: *RI_4C_* = 1 − (*S*/*S* + *U*), where *S* refers to the extent of shared pollinators (i.e. hummingbirds), host tree species or seed dispersers (i.e. lists of shared and unshared species) and *U* refers to the extent of unshared pollinators, host tree species or seed dispersers. The absolute and relative cumulative strength of each barrier were also quantified (calculations also provided in Sobel & Chen (2014)) to determine which barrier is currently contributing the most to maintaining these species in sympatry.

### Statistical analyses

Variation in number of buds per inflorescence, number of flowers per inflorescence, number of fruits per infructescence, flower size measurements (ovary length, anther length, filament length, style length), flower longevity, fruit size measurements (pedicel length and fruit length and width) and accumulated nectar as a function of population was assessed with a generalized linear mixed-effects model (GLMM) in R (R Development Core Team; http://www.r-project.org/). The full GLMM model included population treated as fixed effect and measures as continuous response variables. Plant identity was included in a second model as random effects, and the model with the lowest Akaike information criterion (AIC) was selected as the best model (Akaike, 1981). Nectar replenishment (volume) was analyzed using the same GLMM model, with plant identity and time of day included in a second model as random effects.

Variation in nectar standing crops (volume and mass of sugar in milligrams) among CO, AO, CT populations as a function of time of day was assessed using a general linear model (GLM) in R with Gaussian error and an identity link function. In the model, the effects of population and time of day along with those of the population × time-of-day interaction on nectar volume and mass of sugar in milligrams were assessed using a two-way ANOVA.

To analyze fruit set from the different controlled pollination treatments, binary data were fit to the GLMM model with binomial error and a logit link function, considering the effects of population and controlled pollination treatment as fixed effects and plant identity as random effects. The effects of population and pollination treatment on fruit set were assessed using a two-way ANOVA. A Tukey post-hoc test was used for multiple comparisons among pairs of means.

## Results

### Phylogenetic relationships

**Figure 2:**
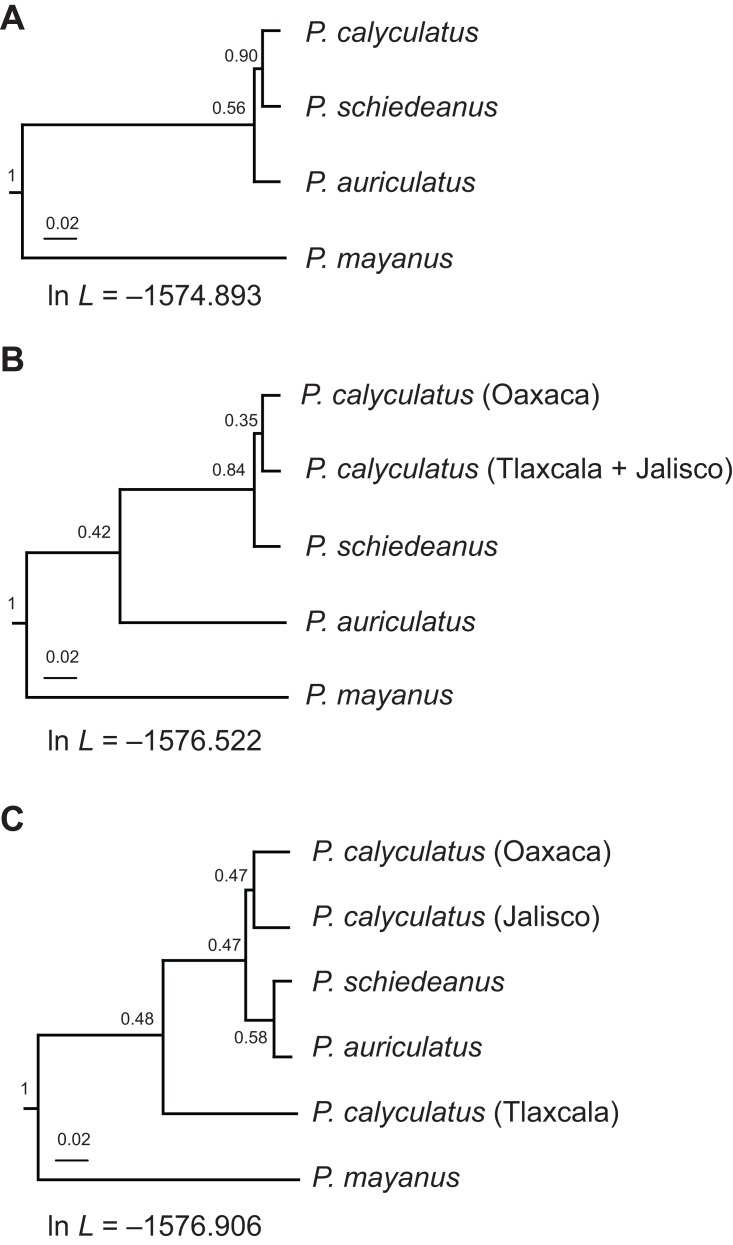
Species delimitation models. (A) *BEAST model with simulation run with all samples of *P. calyculatus* as one lineage (Oaxaca, Tlaxcala, Jalisco), (B) samples of *P. calyculatus* as two lineages corresponding to samples from Santiago Matatlán, Oaxaca and samples from Tlaxcala and Jalisco or (C) three separate lineages (Jalisco, Tlaxcala, Oaxaca). The *BEAST tree of multilocus data for differentiation between populations showed strong support (PP = 0.9) for the monophyly of *P. calyculatus* samples and sister relationship with *P. schiedeanus*. This scenario was the best supported compared with alternative species assignments. The one-species hypothesis produced a higher likelihood score than those for the alternative hypotheses although the difference was not very strong (2 × ln BF; three-species hypothesis versus one-species hypothesis = 4.026; two-species hypothesis versus one-species hypothesis = 3.258; three-species hypothesis versus two-species hypothesis = 0.768).

The *BEAST tree of multilocus data showed strong support for the monophyly of *P. calyculatus* samples and sister relationship with *P. schiedeanus* (PP = 0.9; Fig. 2). This scenario was the best supported compared with alternative species assignments (Fig. 2). Although the difference was not very strong, the one-species hypothesis (*P. calyculatus* samples) produced a higher likelihood score than those for the alternative hypotheses (2 × ln BF; three-species hypothesis versus one-species hypothesis = 4.026; two-species hypothesis versus one-species hypothesis = 3.258; three-species hypothesis versus two-species hypothesis = 0.768). The *BEAST tree analysis supporting the sister relationship between *P. calyculatus* and *P. schiedeanus* samples retrieved a weakly supported relationship between this clade and *P. auriculatus* (Fig. 2).

### Floral and fruit display

In 2013, sympatric *P. calyculatus* and *P. auriculatus* individuals (CO and AO, respectively) were flowering in Oaxaca from August to November, with flowering peaks occurring in August for CO and September for AO. *Psittacanthus calyculatus* in allopatry (CT) bloomed from the end of June through the beginning of November. The 15-tagged individuals followed since August, ended its flowering by October with only few individuals still in bloom. As expected, the highest quantity of fruits was observed at the end of the flowering season.

Total number of buds produced per plant and the number of open flowers per plant was statistically different among populations (Table 1). On average, plants of *P. calyculatus* open more flowers in the allopatric population than those from the other populations, and plants of *P. auriculatus* produce more buds than those of *P. calyculatus*. However, the total number of fruits per plant was not statistically different among populations (Table 1). The GLMM model including plant identity as random effects better fit the data for number of buds, number of open flowers, and number of fruits than the model excluding plant identity (Table 2).

**Table 1:** Floral/fruit displays, flower and fruit measurements, and nectar production for individuals of populations of *Psittacanthus calyculatus* and *P. auriculatus* in sympatry (CO and AO, respectively), and for those from one *P. calyculatus* population in allopatry (CT). Size measurements are expressed in millimeters (mm) and longevity in days (d). Numbers (mean ± SD) with different letters are significantly different after comparisons at P = 0.05 using the adjustment Tukey method.

Variable	CT		CO		AO	
	*n*	Mean ± SD	*n*	Mean ± SD	*n*	Mean ± SD
Number of buds/plant	15	101.9 ± 59.2 a	15	108.6 ± 54.2 a	15	222.3 ± 69.8 b
Number of open flowers/plant	15	31.9 ± 22.9 a	15	12.4 ± 9.1 b	15	6.2 ± 5.4 b
Number of fruits/plant	15	89.5 ± 47.7 a	15	54.4 ± 37.7 a	15	95.1 ± 63.4 a
Ovary length	30	3.92 ± 0.65 a	30	4.20 ± 1.11 a	30	3.32 ± 0.73 b
Anther length	30	3.58 ± 0.32 a	30	4.86 ± 1.03 b	30	7.07 ± 2.28 c
Filament length	30	29.38 ± 4.15 a	30	76.16 ± 19.17 b	30	35.72 ± 11.19 c
Style length	30	33.29 ± 4.31 a	30	83.46 ± 26.58 b	30	41.37 ± 9.69 c
Flower longevity	30	6.27 ± 1.17 a	30	4.77 ± 0.86 b	30	2.43 ± 0.57 c
Nectar volume (μL/flower/day)	20	47.63 ± 20.25 a	20	76.95 ± 37.37 b	20	77.55 ± 35.06 b
Standing crop (μL/flower)	200	9.60 ± 1.24 a	200	10.91 ± 1.36 a	200	4.17 ± 1.65 b
Standing crop (mg/ml/flower)	200	0.18 ± 1.11 a	200	0.23 ± 0.13 a	200	0.17 ± 0.97 b
Replenished nectar (μL/flower)	20	14.65 ± 2.75 a	20	31.32 ± 1.15 a	20	36.46 ± 1.61 a
Pedicel length	53	15.80 ± 1.83 a	53	23.09 ± 2.12 b	53	14.26 ± 1.81 c
Fruit length	53	13.87 ± 0.83 a	53	17.71 ± 0.77 b	53	13.02 ± 0.82 c
Fruit width	53	9.94 ± 0.85 a	53	11.00 ± 0.54 b	53	9.07 ± 0.80 c

**Table 2:** The results of GLMM models for population as fixed effects and plant as random effects from the model affecting the floral, nectar and fruit traits of *Psittacanthus calyculatus* and *P. auriculatus* populations.

Fixed effect	AIC		Estimate	Standard error	χ^2^	Pr (> χ^2^)
	With population identity	Without population identity				
No. of buds/plant	504.74	529.31	6.667	21.195	28.57	< 0.0001
No. of open flowers/plant	375.77	393.09	−19.533	5.324	21.31	< 0.0001
No. of fruits/plant	492.13	494.49	−35.133	17.155	6.35	0.0416
Ovary length	172.98	178.66	−0.297	0.1038	7.68	0.0056
Anther length	180.44	180.44	1.743	0.0805	0	1
Filament length	622.34	623.89	3.170	2.778	0.45	0.0334
Style length	614.39	615.74	4.040	2.927	0.65	0.042
Flower longevity	303.78	391.29	−1.917	0.1502	89.51	< 0.0001
Nectar volume (μL/flower/day)	591.97	599.52	29.325	9.686	11.55	0.0031
Pedicel length	919.58	921.04	−0.773	0.4157	3.46	0.0627
Fruit length	704.51	706.56	−0.426	0.2114	4.05	0.0441
Fruit width	465.28	481.29	−0.432	0.0996	18.01	< 0.0001

**Notes:**

AIC, Akaike information criterion.

### Flower longevity

Flower longevity varied significantly among populations. Floral longevity ranged from 2 to 8 days for all three populations; *P. calyculatus* flowers in allopatry lasted, on average, 2–4 more days than flowers of *P. calyculatus* and *P. auriculatus* in sympatry, respectively (Table 1). Again, the GLMM model including plant identity as random effects better fit the data (Table 2).

### Flower and fruit morphology

Variation in floral morphology among species is shown in Table 1. Among-population differences were statistically significant in most floral measurements, except ovary length in which differences between CT and CO populations were not statistically different. Filament length and style length were significantly larger for flowers from the *P. calyculatus* population in sympatry with *P. auriculatus* than those in allopatry, and anther length was significantly larger and ovary length significantly smaller in *P. auriculatus* (Table 1). The GLMM model including plant identity as random effects better fit the data for ovary length, filament length and style length, but no for anther length (Table 2).

Again, populations were significantly different in fruit size and pedicel length. Fruits from the *P. calyculatus* population in sympatry with *P. auriculatus* were significantly larger and had longer pedicels than those from the allopatric population and the *P. auriculatus* population (Table 1). The GLMM model, as described above, better fit the data for fruit length and fruit width, and marginally better for pedicel length (Table 2).

### Nectar standing crop

**Figure 3:**
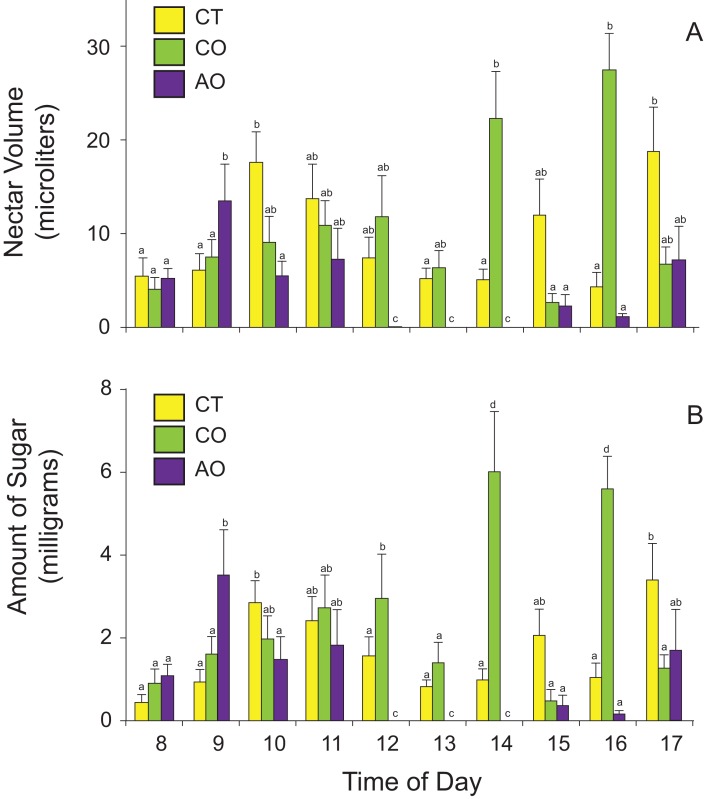
Nectar standing crops for (A) volume and (B) amount of sugar in *Psittacanthus calyculatus* and *P. auriculatus* in sympatry (CO and AO, respectively) and *P. calyculatus* in allopatry (CT) throughout the day. Data are means ± SE.

The amount of nectar available to floral visitors (nectar standing crops) varied among populations and time of day according to the best GLM model (two-way ANOVA, nectar volume: population effect, *F*_2,420_ = 17.79, *P* < 0.0001; time-of-day effect, *F*_9,420_ = 3.16, *P* = 0.001; population × time-of-day interaction, *F*_18,420_ = 6.27, *P* < 0.0001; amount of sugar: population effect, *F*_2,420_ = 12.59, *P* < 0.0001; time-of-day effect, *F*_9,420_ = 3.15, *P* = 0.001; population × time-of-day interaction, *F*_18,420_ = 4.41, *P* < 0.0001). On average, flowers of *P. calyculatus* had more nectar available to floral visitors than flowers of *P. auriculatus* (Table 1). The flowers of *P. calyculatus* had significantly more nectar left at noon than the flowers of *P. auriculatus* in sympatry, and these differences in nectar standing crop were more evident in the afternoon (Fig. 3).

### Nectar secretion pattern

Undisturbed flowers of *P. calyculatus* and *P. auriculatus* in sympatry accumulate ∼77 microliters per flower after 24 h of accumulation but allopatric *P. calyculatus* flowers accumulate about half, and these differences were statistically significant (Table 1). The GLMM model with population as fixed effects and plant identity as random effects better fit the data for accumulated nectar volume (Table 2).

Nectar replenishment after repeated removal varied significantly among populations (two-way ANOVA, nectar volume: population effect, *F*_2,162_ = 20.52, *P* < 0.0001; time-of-day effect, *F*_2,120_ = 4.38, *P* = 0.0139; population × time-of-day interaction, *F*_4,162_ = 5.56, *P* = 0.0003). On average, plants of *P. calyculatus* and *P. auriculatus* in sympatry secreted more nectar after repeated removals than *P. calyculatus* flowers in allopatry (Table 1). The GLMM model with population and time of day as fixed effects and plant identity as random effects better fit the data (AIC = 1,554.2) than the model excluding plant identity (AIC = 1,629.5; Likelihood-ratio test = 87.28, *P* < 0.0001).

### Floral pollinators and fruit consumers

A total of 155 visits from nine hummingbird species were registered during our observations (CT = 41, CO = 55, AO = 59; four to six hummingbird species at each population). The most common hummingbird species, *Amazilia beryllina*, was shared between sympatric *P. calyculatus* and *P. auriculatus* (61.8% and 66.1% of total visits, respectively), followed by *Cynanthus sordidus* (30.9%) and *Calothorax pulcher* (13.5%), respectively. Other less common hummingbird visits accounted for the remaining visits (Table 3). In allopatry, the most common hummingbird species on *P. calyculatus* flowers was *Eugenes fulgens* (34% of total visits), followed by *A. beryllina* (26.8%), *Cynanthus latirostris* (19.5%) and *Calothorax lucifer* (12.2%). Hummingbirds were active throughout the day, whereas insect foraging activity was more restricted to warmer hours.

**Table 3:** Floral visitors (A) and fruit consumers (B) for *Psittacanthus calyculatus* and *P. auriculatus* in sympatry (CO and AO, respectively) and for *P. calyculatus* in allopatry (CT).

(A) Floral visitors			
Order	CT	CO	AO
Lepidoptera	*Papilio multicaudatus*	*Papilio multicaudatus*	*Papilio multicaudatus*
		*Phocides urania*	*Phocides urania*
		*Anteos clorinde*	*Anteos clorinde*
		*Phoebis sennae*	*Phoebis sennae*
Hymenoptera	*Apis mellifera*	*Apis mellifera*	*Apis mellifera*
	*Bombus* sp.	*Bombus* sp.	*Bombus* sp.
	Vespidae	Vespidae	Vespidae
Apodiformes	*Colibri thalassinus* (2.4%)		
	*Hylocharis leucotis* (4.9%)		
	*Cynanthus latirostris* (19.5%)		*Cynanthus latirostris* (5.1%)
		*Cynanthus sordidus* (30.9%)	*Cynanthus sordidus* (8.4%)
	*Amazilia beryllina* (26.8%)	*Amazilia beryllina* (61.8%)	*Amazilia beryllina* (66.1%)
		*Amazilia violiceps* (5.4%)	*Amazilia violiceps* (6.8%)
	*Eugenes fulgens* (34.1%)		
	*Calothorax lucifer* (12.2%)		
		*Calothorax pulcher* (1.8%)	*Calothorax pulcher* (13.5%)

(B) Seed dispersers			
Order	CT	CO	AO
Passeriformes	*Tyrannus vociferans* (58.8%)		*Tyrannus vociferans* (77.8%)
		*Myiozetetes similis* (66.7%)	
		*Pitangus sulphuratus* (4.2%)	
	*Ptilogonys cinereus* (29.4%)	*Ptilogonys cinereus* (8.3%)	*Ptilogonys cinereus* (16.7%)
	*Icterus* sp. (5.9%)	*Icterus cucullatus* (20.8%)	*Icterus cucullatus* (5.6%)
	*Turdus rufopalliatus* (5.9%)		

**Notes:**

Numbers in parentheses are percentages of total number of visits in a population.

Honeybees (*Apis mellifera*), bumble bees (*Bombus*), wasps (Vespidae) and butterflies (*Papilio*, *Phocides*, *Anteos*, *Phoebis*) also visited mistletoe flowers during our focal observations (Table 3; Fig. 1), but their frequency was not quantified as previously explained.

For seed dispersers, during our observations we registered a total of 118 visits of different bird species (CT = 34, CO = 48, AO = 36). Interestingly, the observed species assemblage of fruit consumers differed between the two *Psittacanthus* species in sympatry. The most common fruit consumer of *P. calyculatus* (CO) was *Myiozetetes similis* (66.7% of total visits), followed by *Icterus cucullatus* (20.8%), whereas for *P. auriculatus* (AO) the most common was *Tyrannus vociferans* (77.8% of total visits) followed by *Ptilogonys cinereus* (16.7%). For *P. calyculatus* in allopatry (CT), *T. vociferans* (58.8% of total visits) was also the most common fruit consumer, followed by *P. cinereus* (29.4%). Other bird species accounted for the remaining visits (Table 3).

### Breeding system

Anther’s dehiscence on flowers of both mistletoe species occurred on day 1. In *P. auriculatus*, stigmas were most receptive on day 1 flowers, whereas for *P. calyculatus* higher receptivity of stigmas was observed until day 3 from bud opening.

**Figure 4:**
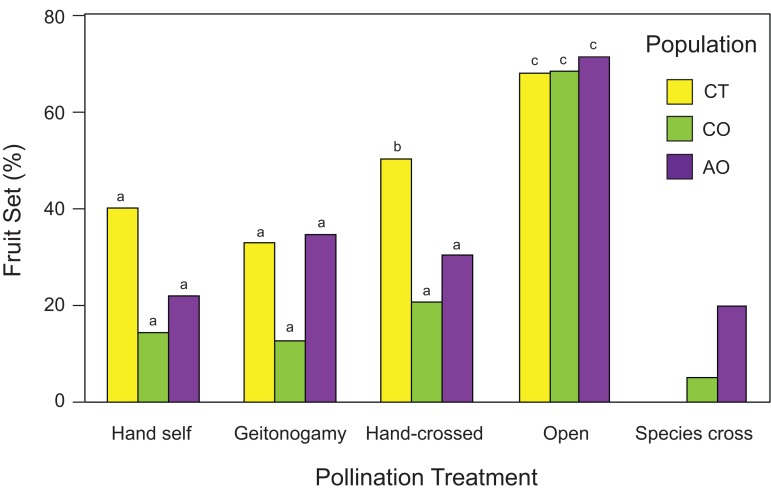
Fruit set (number of fruit/number of flowers) of *Psittacanthus calyculatus* and *P. auriculatus* in sympatry (CO and AO, respectively) and *P. calyculatus* in allopatry (CT) by pollination treatment. Reciprocal crosses (interspecific pollination) were performed between sympatric populations of *P. calyculatus* and *P. auriculatus*.

Flowers from all pollination treatments set fruit; both hand-crossed (xenogamy) flowers set fruits as well as flowers exposed to geitonogamous and hand-self crosses, and flowers exposed to open pollination (Fig. 4). However, the probability of fruit production was not independent of population and pollination treatment according to the best GLM model (two-way ANOVA; population effects: χ^2^ = 8.96, df = 2, *P* = 0.0113; pollination treatment effects: χ^2^ = 43.58, df = 3, *P* < 0.0001; population × pollination treatment interaction: χ^2^ = 5.62, df = 6, *P* = 0.0466), with higher fruit production among flowers of all three populations exposed to open pollination (> 60% in all cases) followed by hand-crossed *P. calyculatus* flowers (CT and CO) and geitonogamous *P. auriculatus* flowers (Fig. 4). One caveat about the interpretation of the pollination experiment needs to be mentioned, however: all hand pollinations resulted in lower fruit set than the natural pollinations, indicating that hand-pollinated flowers are not showing the true potential to produce seeds of which they are capable given the available resources.

### Reproductive isolation

Interspecific crosses between species did produce fruit (Fig. 4), indicating pollen compatibility between the *Psittacanthus* species. However, fruit set in flowers exposed to interspecific pollination was two times higher from *P. calyculatus* to *P. auriculatus* (20%) than in the opposite direction (5%; Fig. 4).

Using *RI* estimate *RI_4A_*, there were fewer heterospecific matings than expected by chance in *P. calyculatus* (*RI_4A_* = 0.629) as compared to *P. auriculatus* (*RI_4A_* = 0.20). When considering other factors of ecological isolation that affect co-occurrence, the *RI* values for isolation by hummingbird pollinators, host tree species, and seed dispersers using *RI_4C_* were 0.20, 0.80 and 0.60, respectively, suggesting that host usage is the most important ecological isolation factor between the two species (Table 4). Accordingly, the absolute and relative cumulative strength values indicate that the host tree species’ barrier is currently contributing the most to maintaining these species in sympatry (Table 4).

**Table 4:** Contribution of assessed pre-pollination (A) and post-pollination (B) barriers to reproductive isolation (*RI*) between *P. calyculatus* (CO) and *P. auriculatus* (AO) in sympatry for the studied reproductive barriers. Isolation components generally vary from zero (no barrier) to one (complete isolation). Contributions to total *RI* were calculated for sequential reproductive barriers, with the sum of contributions equaling total isolation. Sobel & Chen’s (2014) methodology was used for *RI* (see Supplemental Information for detailed explanations of *RI* calculations).

Isolating barrier	Raw values		Sobel & Chen (2014) *RI* value	Absolute cumulative strength	Relative cumulative strength
(A) Pre-pollination barriers affecting co-occurrence	Heterospecific	Conspecific			
Host species isolation	0.200	0.800	0.800	0.800	0.808
Seed dispersers isolation	0.400	0.600	0.600	0.120	0.121
Pollinator isolation	0.800	0.200	0.200	0.016	0.016
(B) Post-pollination barriers not affecting co-occurrence	Shared	Unshared			
CO Fruit set	0.185	0.814	0.629	0.049	0.049
AO Fruit set	0.400	0.600	0.200	0.005	0.005
**Total isolation**	**0.990**				

## Discussion

As expected for species that overlap geographically, we observed differences in floral traits, nectar production dynamics and floral visitors, and seed dispersers and host usage between populations of closely related *Psittacanthus calyculatus* and *P. auriculatus* in sympatry. Despite their floral trait differences and contrary to expectations of pollinator-driven speciation models, we found low pollinator specificity, suggesting that pollinator isolation is here a weak barrier between these species. Other *RI* mechanisms including those related to differences in host usage, or potential post-mating barriers that were not evaluated, might be acting as barriers to prevent hybridization in the absence of high pollinator specificity. These aspects are further discussed below.

### Phylogenetic relationships

A first molecular phylogeny of several *Psittacanthus* species showed monophyly of *Psittacanthus* species (Ornelas et al., 2016), and that the clade involving *P. calyculatus*, *P. schiedeanus* and *P. auriculatus* originated less than 2.8 million years ago. Samples of *P. calyculatus* and *P. schiedeanus* turned out to be monophyletic, but little support and genetic structure was observed within this clade. Here, the *BEAST tree analysis distinguished two resolved clades supported by high PP values. One included all samples of *P. calyculatus* from Jalisco, Tlaxcala and Oaxaca and the other is composed of *P. schiedeanus* samples, all of which produce yellow-to-orange flowers. *Psittacanthus auriculatus* with pink-to-red flowers was retrieved as the sister lineage of the *P. calyculatus*/*P. schiedeanus* clade. This one-species hypothesis (*P. calyculatus* populations) scenario was the best supported compared with alternative population assignments, suggesting that post-mating barriers evolved during divergence between *P. calyculatus*/*P. schiedeanus* and *P. auriculatus*.

The scenario for genetic differentiation between *P. calyculatus* populations from Tlaxcala and Oaxaca using two loci was not supported by the *BEAST tree analysis. However, distributions of the two focal groups of *P. calyculatus* display a pattern of isolation by altitude and habitat type, with a sympatric region shared with *P. auriculatus* in more xeric conditions in Oaxaca and a more temperate habitat at higher elevation in Tlaxcala extending to western of the Trans-Mexican Volcanic Belt. We also found significant trait differences between the two populations of *P. calyculatus*. For instance, plants in sympatry with *P. auriculatus* (CO) produced flowers with longer filaments and styles than those of the allopatric population (CT). The mean filament length in CO is over twice that of CT (71.34 vs. 29.38 mm), and this exceeds the dimensions reported by Kuijt (2009) for this species (range 25–50 mm) and by Azpeitia & Lara (2006) for another population in Tlaxcala (range 37–41 mm). The observed trait divergence between populations of *P. calyculatus* could be attributed to (a) typical variation in floral traits between populations, given the considerable variation between populations in this taxon throughout its range according to Kuijt (2009), or (b) resulted from changes regarding the local communities of pollinators, and, therefore, expected for peripheral, allopatric populations or (c) evolved in the sympatric population to reduce interspecific competition with *P. auriculatus* for pollinators. A more detailed sampling of *P. calyculatus* along the Trans-Mexican Volcanic Belt and sample genotyping using microsatellites will be useful to assess whether the sympatric population may be a different undescribed species and to test whether these differences in environmental conditions could contribute strongly to the *RI* between populations of *P. calyculatus*.

### Floral biology and nectar

Morphologically, the two *Psittacanthus* species in sympatry differ in some floral traits that could be important in determining the potential of one species to influence the pollination of the other. In particular, the filaments are extremely long and spread out and no discernable floral tube is formed in *P. calyculatus* flowers (Azpeitia & Lara, 2006). In contrast, the flowers with curled petals and clearly forming a floral tube are protandrous in *P. auriculatus* (Pérez-Crespo et al., 2016). At opening, stigma and anthers of *P. auriculatus* remain erect in close proximity, and in subsequent hours, the style begins to elongate and the stigma separates upwards (for further details see Pérez-Crespo et al., 2016). The elongation of style and anther/stigma separation could facilitate the contact of stigma with the hummingbird’s head during the second day of higher stigma receptivity, promoting cross-pollen transfer and avoiding self-interference (see also Pérez-Crespo et al., 2016). In general, the floral characteristics of *P. calyculatus* and *P. auriculatus* fit the pollination syndrome of ornithophily within Loranthaceae (Faegri & Van der Pijl, 1979*;* Calder & Bernhardt, 1984; Azpeitia & Lara, 2006; Pérez-Crespo et al., 2016), but their differences in flower morphology evoke the existence of different hummingbird-pollination syndromes.

The more extended flowering period of *P. calyculatus* (∼5 months), as reported in a different population (Azpeitia & Lara, 2006), than the observed for *P. auriculatus* (∼2 months; Pérez-Crespo et al., 2016) may affect *RI* of sympatric species (e.g., Savolainen et al., 2006). We did not quantify their flowering phenology in sympatry, but the extensive overlap in their flowering times contributes little to pre-pollination *RI* in this case. Interestingly, we found that flowers of *P. calyculatus* in sympatry produced more nectar and the plants produced more flowers, resulting in a larger display. It is possible that these differences may be a result of competition for pollinators, but more rigorous measurements of flower offer along the blooming season would be necessary.

### Shared pollinators

In populations of the two examined sympatric species we observed interspecific visitations of hummingbirds, suggesting that pollinators in general do not completely discriminate between their flowers. The most frequent floral visitor, *Amazilia beryllina*, was shared by both *Psittacanthus* sympatric populations (61–66% of all hummingbird visits), although the other hummingbird species that were shared, *Cynanthus sordidus*, varied in their visitation frequency. Thus, pollinator discrimination and/or temporal variation in the composition of the pollinator species assemblage might contribute little to pre-pollination isolation between the two sympatric species.

While hummingbirds presumably increased seed production of open-pollinated flowers in both species, differences in their foraging behavior on mistletoe flowers and other aspects of the plants’ reproductive biology such as the spatial and temporal separation of the androecium and gynoecium (e.g., protandry in *P. auriculatus*; Pérez-Crespo et al., 2016), may also affect relevant aspects of gene flow that we did not measure such as the transfer of pollen grains among conspecific plants (Tadey & Aizen, 2001; Kuijt, 2009). For instance, territorial hummingbird foraging behavior on flowers of *P. robustus* seems to favor geitonogamy because they probed many flowers per plant, suggesting that the non-territorial traplining hummingbird species may play a role in promoting long-distance gene flow in the *P. robustus* pollination system (Guerra, Galetto & Silva, 2014).

Interestingly, we found that nectar standing crops differed between sympatric populations, with the peak time of day shifted to the afternoon in *P. calyculatus*, as if avoiding interspecific pollinator movements. We did not quantify visitation rates of all floral visitors to assess whether *P. calyculatus* plants with more open flowers (i.e., larger floral displays) or individual flowers were more often visited than those of *P. auriculatus*, or tracked individual hummingbirds to assess interspecific pollen movement. However, nectar standing crops (nectar available in flowers throughout the day) suggest that both *Psittacanthus* species are similarly visited during the early hours but in the afternoon *P. calyculatus* flowers were less often visited to then being more often visited towards the end of day. Given the larger number of open flowers in *P. calyculatus* plants and the observed nectar standing crop shifts in the afternoon, we hypothesize that *P. calyculatus* flowers were more often visited by hummingbirds that carry more pollen of the species to *P. auriculatus* stigmas, leading to the possibility of asymmetric hybridization.

Despite the slight differences in hummingbird pollinators, differential pollinator attraction via floral trait differences does not appear to be the primary barrier between these species. Further quantitative data on foraging behavior and effectiveness of each floral visitor type are needed to test whether *RI* has involved the floral traits and pollination ecology between the two sympatric populations. Beyond quantifying interspecific pollinator movement, future work should also examine pollen loads to determine whether shared hummingbird pollinators are equally effective at pollen removal/deposition for each species/population of mistletoes, and whether interspecific pollen flow is substantial and asymmetric.

### Breeding system and interspecific compatibility

Flowers of both *Psittacanthus* species are not apomictic and to some extent self-compatible, although open pollination resulted in more fruits. Like in many bird-pollinated members of Loranthaceae with hermaphroditic flowers, the *Psittacanthus* species are predominantly outbreeding plants, even though self-pollination can be effective (Azpeitia & Lara, 2006; Ramírez & Ornelas, 2010; Guerra, Galetto & Silva, 2014; Pérez-Crespo et al., 2016). The pre-pollination mechanisms are considered to be more important generally than post-pollination mechanisms in the *RI* of sympatric populations, because theoretically they should reduce gamete waste more strongly, and discriminative floral visitation and floral constancy by pollinators are believed to be the main pre-pollination isolation’ mechanisms (e.g., Ramsey, Bradshaw & Schemske, 2003; Kay, 2006).

Interspecific crosses produced fruit at the study site, indicating pollen compatibility between the *Psittacanthus* species. This is not surprising given the relatively recent origin of these species (Ornelas et al., 2016), suggesting that post-mating isolation mechanisms are still evolving. Fruit set was higher when flowers of *P. auriculatus* were pollinated with *P. calyculatus* pollen grains than in the opposite direction, though fruit set after interspecific cross pollination was lower than intraspecific pollination for both mistletoe species. A breakdown in the mating system associated with modifications and divergence in floral traits that would promote *RI* to minimize competition was expected because these co-occurring plants share the same habitat and the most frequent hummingbird pollinators, and their flowering times overlap extensively (e.g., DeWitt Smith et al., 2008; Palma-Silva et al., 2011; Briscoe Runquist & Moeller, 2014; Carrió & Güemes, 2014; and references therein). The higher fruit/seed set of *P. calyculatus* in crosses with *P. auriculatus* is expected in crosses between plants in which outcrossing parents “overpower” selfing parents (the weak inbreeder/strong outbreeder WISO hypothesis; Brandvain & Haig, 2005) and, therefore, the outcrossing differences between species likely contributing to post-mating barriers do not fully explain our data.

Mechanisms by which the mating system could act as a *RI* barrier are largely unknown in plants (e.g., Palma-Silva et al., 2011; Armbruster, 2014). Interspecific fruit/seed production between the studied *Psittacanthus* species suggests the potential for hybridization. However, interspecific fruit/seed production was lower compared to intraspecific fruit/seed production in control plants, indicating the presence of post-mating barriers to prevent hybrid formation. Ongoing work based on nuclear microsatellite markers will help to understand if the potential for hybridization between these congeneric species is related to the breaking of pollination barriers and asymmetric introgression towards *P. auriculatus*.

### Consequences of sympatry and the potential for host specialization

We found that the most frequent avian seed disperser was different between species as well as the host tree species composition and its prevalence of infection, suggesting the potential for differential local specialization on host tree species. In the area of sympatry, *P. calyculatus* mistletoes were growing mainly on *Celtis caudata* trees and *P. auriculatus* mistletoes on *Acacia schaffneri* trees. Larger fruits of *P. calyculatus* were most frequently consumed by *Myiozetetes similis*, followed by *Icterus cucullatus*, whereas smaller fruits of *P. auriculatus* were most frequently consumed by *Tyrannus vociferans* followed by *Ptilogonys cinereus*.

It is possible that the differences between *Psittacanthus* species in the preferences between most frequent seed dispersers are linked to the higher number of fruits per plant in *P. auriculatus*. It is also possible that the observed differences in host use and prevalence of infection are linked to species differences in host compatibility and/or to differences in foraging behavior and defecation patterns of bird dispersers and seed deposition of *P. calyculatus* and *P. auriculatus* mistletoe seeds. Although high specialization in some *Psittacanthus* species may be the result of frequent encounters between mistletoe seeds and the commonest host plants (host abundance) and that compatibility between mistletoes and tree species is only a pre-condition for mistletoe-host parasitism (Fadini, 2011), few studies have argued that non-random perch preferences of bird seed dispersers, along with their foraging behavior differences and post-foraging movements, are also important for shaping the patterns of mistletoe infection prevalence and, ultimately, determining host specificity (Monteiro, Martins & Yamamoto, 1992; López de Buen & Ornelas, 1999). The decisions taken by birds may either result in a pattern that concentrates mistletoe seeds on the most abundant trees (López de Buen & Ornelas, 1999), or even on different and the less abundant ones (Aukema & Martínez del Rio, 2002; Roxburgh & Nicolson, 2005).

We find that the measured reproductive barriers are sufficient to cause nearly complete *RI* between the two study species. By calculating the sequential contributions of pre- and post-pollination barriers (sensu Baack et al., 2015) to gene flow following the Sobel & Chen’s (2014) methodology, we compute the total *RI* between *P. auriculatus* and *P. calyculatus* to be 0.990. The total isolation achieved in nature is probably higher than this value because several components of *RI* were not studied (phenological isolation, efficiency of pollinators in cross-species flower visitation, F1 survivorship, F2 hybrid breakdown) or because the contributions of some barriers were estimated conservatively (see also Ramsey, Bradshaw & Schemske, 2003). Nonetheless, the cumulative strength of pre-pollination barriers in *P. auriculatus* and *P. calyculatus* greatly outweighs those of post-pollination barriers (Table 4). Future work should consider the position of mistletoe infections, the relative abundance of host tree species and surveys of mistletoe seed deposition, and post-foraging observations documenting movements of potential bird dispersers within the study area to determine whether these seed dispersers are sufficiently specialized to isolate mistletoe populations on different hosts, as suggested by the absolute and relative cumulative strength values of this barrier.

## Conclusions

Collectively, our data show marked trait divergence between populations considered as *P. calyculatus* and between populations of closely related *P. auriculatus* and *P. calyculatus* in sympatry. Although floral traits in *P. calyculatus* appear to be very plastic, future studies should investigate whether the observed differences in floral morphology between allopatric populations is simply a by-product of adaptation to pollination environments that differ between the allopatric and sympatric portions of the species’ range or is the result of interactions with congeners in sympatry. For populations of *P. calyculatus* and *P. auriculatus* that overlap geographically, we observed accentuated differences in floral traits, but differential pollinator attraction via floral trait differences does not appear to be the primary isolation barrier between these species. Instead, host usage seem to currently contributing the most to maintaining these species in sympatry. If pollinators are transporting inter-specific pollen, however, other post-mating barriers to prevent hybridization are likely present because interspecific fruit/seed set was highly reduced. Our study represents a first view of the breeding system and pollination ecology of these two *Psittacanthus* species in sympatry to determine the absolute and relative strength of several ecological factors in their total *RI*, in which simple shifts in host usage mediated by seed dispersers seem to be primary drivers of mistletoe speciation rather than shifts in floral traits mediated by pollinator selection.

## Supplemental Information

10.7717/peerj.2491/supp-1 Supplemental Information 1 Voucher information of the Psittacanthus populations used in the study. IDs reported below refer to accession numbers in the Instituto de Ecología, AC (XAL) herbarium.

## Additional Information and Declarations

### Field Study Permissions

The following information was supplied relating to field study approvals (i.e., approving body and any reference numbers): We obtained collecting permits from the Mexican government to conduct this work from the Secretaría de Medio Ambiente y Recursos Naturales, Instituto Nacional de Ecología, Dirección General de Vida Silvestre (permit number: (INE, SEMARNAT, SGPA/DGGFS/712/1299/12). The collecting permit specifically allowed for the collection of leaf tissue samples described in this study as newly sequenced. We had access to the material under the terms of the scientific permit and no specific permits for fieldwork were required to work at the study areas though municipal and community authorities of Nativitas and Tetlatlahuca, Tlaxcala, and Santiago Matatlán, Oaxaca were informed. Leaf tissue samples were obtained from the plant species reported here with no further manipulation.

### DNA Deposition

The following information was supplied regarding the deposition of DNA sequences: All sequences of *P. calyculatus* and *P. auriculatus* and those new of other *Psittacanthus* species have been submitted to GenBank (Accession nos. *ITS*: KX241619–KX241734, *trnL-F*: KX241735–KX241823). Voucher information of the *Psittacanthus* populations used in the study is given in Table S1.

### Data Deposition

The following information was supplied regarding data availability: Díaz Infante S, Lara C, Arizmendi MC, Eguiarte LE, Ornelas JF. 2016. Data from: Reproductive ecology and isolation of *Psittacanthus calyculatus* and *P. auriculatus* mistletoes (Loranthaceae). Dryad Digital Repository. DOI: 10.5061/dryad.6kt60.

## References

[ref-1] Acosta PR, Cházaro BM, Patiño BRM (1993). Los muérdagos (Loranthaceae) del estado de Tlaxcala, México.

[ref-2] Akaike H (1981). Likelihood of a model and information criteria. Journal of Econometrics.

[ref-3] Arce I (2012). Factores bióticos asociados a la distribución de *Psittacanthus calyculatus* en la zona periurbana de la ciudad de Querétaro, México.

[ref-4] Armbruster WS (2014). Floral specialization and angiosperm diversity: phenotypic divergence, fitness trade-offs and realized pollination accuracy. AoB Plants.

[ref-5] Arruda R, Fadini RF, Carvalho LN, Del-Claro K, Mourão FA, Jacobi CM, Teodoro GS, van den Berg E, Caires CS, Dettke GA (2012). Ecology of Neotropical mistletoes: an important canopy-dwelling component of Brazilian ecosystems. Acta Botanica Brasilica.

[ref-6] Aukema JE, Martínez del Rio C (2002). Variation in mistletoe seed deposition: effects of intra- and interspecific host characteristics. Ecography.

[ref-7] Azpeitia F, Lara C (2006). Reproductive biology and pollination of the parasitic plant *Psittacanthus calyculatus* (Loranthaceae) in central Mexico. Journal of the Torrey Botanical Society.

[ref-8] Baack E, Melo MC, Rieseberg LH, Ortiz-Barrientos D (2015). The origins of reproductive isolation in plants. New Phytologist.

[ref-9] Brandvain Y, Haig D (2005). Divergent mating systems and parental conflict as a barrier to hybridization in flowering plants. American Naturalist.

[ref-10] Briscoe Runquist RD, Moeller DA (2014). Floral and mating system divergence in secondary sympatry: testing an alternative hypothesis to reinforcement in *Clarkia*. Annals of Botany.

[ref-11] Brown WL, Wilson EO (1956). Character displacement. Systematic Zoology.

[ref-12] Brys R, Broeck AV, Mergeay J, Jacquemyn H (2014). The contribution of mating system variation to reproductive isolation in two closely related *Gentaurium* species (Gentianaceae) with a generalized flower morphology. Evolution.

[ref-13] Calder M, Bernhardt P (1984). The Biology of Mistletoes.

[ref-14] Calderón de Rzedowski G, Rzedowski J (2002). Flora fanerogámica del Valle de México.

[ref-15] Carrió E, Güemes J (2014). The effectiveness of pre- and post-zygotic barriers in avoiding hybridization between two snapdragons (*Antirrhinum* L.: Plantaginaceae). Botanical Journal of the Linnean Society.

[ref-16] Coyne JA, Orr HA (2004). Speciation.

[ref-17] DeWitt Smith S, Hall SJ, Izquierdo PR, Baum DA (2008). Comparative pollination biology of sympatric and allopatric Andean *Iochroma* (Solanaceae). Annals of the Missouri Botanical Garden.

[ref-18] Doyle JJ, Doyle JL (1987). A rapid DNA isolation procedure from small quantities of fresh leaf tissue. Phytochemical Bulletin.

[ref-19] Drummond AJ, Rambaut A (2007). BEAST: Bayesian evolutionary analysis by sampling trees. BMC Evolutionary Biology.

[ref-20] Fadini RF (2011). Non-overlap of hosts used by three congeneric and sympatric loranthaceous mistletoe species in an Amazonian savanna: host generalization to extreme specialization. Acta Botanica Brasilica.

[ref-21] Faegri K, Van der Pijl L (1979). The Principles of Pollination Ecology.

[ref-22] Grossenbacher D, Briscoe Runquist RD, Goldberg EE, Brandvain Y (2016). No association between plant mating system and geographic range overlap. American Journal of Botany.

[ref-23] Grossenbacher DL, Whittall JB (2011). Increased floral divergence in sympatric monkeyflowers. Evolution.

[ref-24] Guerra TJ, Galetto L, Silva WR (2014). Nectar secretion dynamic links pollinator behavior to consequences for plant reproductive success in the ornithophilous mistletoe *Psittacanthus robustus*. Plant Biology.

[ref-25] Heled J, Drummond AJ (2010). Bayesian inference of species trees from multilocus data. Molecular Biology and Evolution.

[ref-26] Hopkins R (2013). Reinforcement in plants. New Phytologist.

[ref-27] Jang Y, Gerhardt HC (2006). Divergence in the calling songs between sympatric and allopatric populations of the southern wood cricket *Gryllus fultoni* (Orthoptera: Gryllidae). Journal of Evolutionary Biology.

[ref-28] Johnson MA, Price DK, Price JP, Stacy EA (2015). Postzygotic barriers isolate sympatric species of *Cyrtandra* (Gesneriaceae) in Hawaiian montane forest understories. American Journal of Botany.

[ref-29] Kay KM (2006). Reproductive isolation between two closely related hummingbird-pollinated Neotropical gingers. Evolution.

[ref-30] Kay KM, Whittall JB, Hodges SA (2006). A survey of nuclear ribosomal internal transcribed spacer substitution rates across angiosperms: an approximate molecular clock with life history effects. BMC Evolutionary Biology.

[ref-31] Kearns CN, Inouye DW (1993). Techniques for Pollination Biologists.

[ref-32] Kuijt J (2009). Monograph of Psittacanthus (Loranthaceae).

[ref-33] Kuijt J (2014). Five new species, one new name, and transfers in Neotropical mistletoes (Loranthaceae), miscellaneous notes, 61–68. Novon: A Journal for Botanical Nomenclature.

[ref-34] Lara C, Pérez G, Ornelas JF (2009). Provenance, guts, and fate: field and experimental evidence in a host-mistletoe-bird system. Ecoscience.

[ref-35] Leal FC, Lopes AV, Machado IC (2006). Polinização por beija-flores em uma área de caatinga no Município de Floresta, Pernambuco, Nordeste do Brasil. Revista Brasileira de Botânica.

[ref-36] López de Buen L, Ornelas JF (1999). Frugivorous birds, host selection and the mistletoe *Psittacanthus schiedeanus*, in central Veracruz, Mexico. Journal of Tropical Ecology.

[ref-37] Lowry DB, Modliszewski JL, Wright KM, Wu CA, Willis JH (2008). The strength and genetic basis of reproductive isolating barriers in flowering plants. Philosophical Transactions of the Royal Society B: Biological Sciences.

[ref-38] Mathiasen RL, Nickrent DL, Shaw DC, Watson DM (2008). Mistletoes: pathology, systematics, ecology, and management. Plant Disease.

[ref-39] Monteiro RF, Martins RP, Yamamoto K (1992). Host specificity and seed dispersal of *Psittacanthus robustus* (Loranthaceae) in south-east Brazil. Journal of Tropical Ecology.

[ref-40] Montgomery BR, Kelly D, Robertson AW, Ladley JJ (2003). Pollinator behavior, not increased resources, boosts seed set on forest edges in a New Zealand Loranthaceous mistletoe. New Zealand Journal of Botany.

[ref-41] Norton DA, Carpenter MA (1998). Mistletoes as parasites: host specificity and speciation. Trends in Ecology & Evolution.

[ref-42] Ornelas JF, Gándara E, Vásquez-Aguilar AA, Ramírez-Barahona S, Ortiz-Rodriguez AE, González C, Mejía Saules MT, Ruiz-Sanchez E (2016). A mistletoe tale: postglacial invasion of *Psittacanthus schiedeanus* (Loranthaceae) to Mesoamerican cloud forests revealed by molecular data and species distribution modeling. BMC Evolutionary Biology.

[ref-43] Palma-Silva C, Wendt T, Pinheiro F, Barbará T, Fay MF, Cozzolino S, Lexer C (2011). Sympatric bromeliad species (*Pitcairnia* spp.) facilitate tests of mechanisms involved in species cohesion and reproductive isolation in Neotropical inselbergs. Molecular Ecology.

[ref-44] Pascarella JB (2007). Mechanisms of prezygotic reproductive isolation between two sympatric species, *Gelsemium rankinii* and *G. sempervirens* (Gelsemiaceae), in the southeastern United States. American Journal of Botany.

[ref-45] Pérez-Crespo MJ, Ornelas JF, Martén-Rodríguez S, González-Rodríguez A, Lara C (2016). Reproductive biology and nectar production of the Mexican endemic *Psittacanthus auriculatus* (Loranthaceae), a hummingbird-pollinated mistletoe. Plant Biology.

[ref-46] Posada D (2008). jModelTest: phylogenetic model averaging. Molecular Biology and Evolution.

[ref-47] Ramírez MM, Ornelas JF (2010). Pollination and nectar production of *Psittacanthus schiedeanus* (Loranthaceae) in central Veracruz, Mexico. Boletín de la Sociedad Botánica de México.

[ref-48] Ramsey J, Bradshaw HD, Schemske DW (2003). Components of reproductive isolation between the monkeyflowers *Mimulus lewisii* and *M. cardinalis* (Phrymaceae). Evolution.

[ref-49] Richardson JE, Pennington RT, Pennington TD, Hollingsworth PM (2001). Rapid differentiation of a species-rich genus of Neotropical rain forest trees. Science.

[ref-50] Rieseberg LH, Willis JH (2007). Plant speciation. Science.

[ref-51] Robertson AW, Kelly D, Ladley JJ, Sparrow AD (1999). Effects of pollinator loss on endemic New Zealand mistletoes (Loranthaceae). Conservation Biology.

[ref-52] Roxburgh L, Nicolson SW (2005). Patterns of host use in two African mistletoes: the importance of mistletoe-host compatibility and avian disperser behaviour. Functional Ecology.

[ref-53] Savolainen V, Anstett M-C, Lexer C, Hutton I, Clarkson JJ, Norup MV, Powell MP, Springate D, Salamin N, Baker WJ (2006). Sympatric speciation in palms on an oceanic island. Nature.

[ref-54] Schluter D, McPhail JD (1992). Ecological character displacement and speciation in sticklebacks. American Naturalist.

[ref-55] Sobel JM, Chen GF (2014). Unification of methods for estimating the strength of reproductive isolation. Evolution.

[ref-56] Suh Y, Thien LB, Reeve HE, Zimmer EA (1993). Molecular evolution and phylogenetic implications of internal transcribed spacer sequences of ribosomal DNA in Winteraceae. American Journal of Botany.

[ref-57] Taberlet P, Gielly L, Pautou G, Bouvet J (1991). Universal primers for amplification of three non-coding regions of chloroplast DNA. Plant Molecular Biology.

[ref-58] Tadey M, Aizen MA (2001). Why do flowers of a hummingbird-pollinated mistletoe face down?. Functional Ecology.

[ref-59] Watson DM (2001). Mistletoe—a keystone resource in forests and woodlands worldwide. Annual Review of Ecology and Systematics.

[ref-60] White TJ, Burns T, Lee S, Taylor J, Innis MA, Gelfand DH, Sninsky JJ, White TJ (1990). Amplification and direct sequencing of fungal ribosomal RNA genes for phylogenetics. PCR Protocols: A Guide to Methods and Applications.

[ref-61] Widmer A, Lexer C, Cozzolino S (2009). Evolution of reproductive isolation in plants. Heredity.

[ref-62] Zuria I, Castellanos I, Gates JE (2014). The influence of mistletoes on birds in an agricultural landscape of central Mexico. Acta Oecologica.

