# Supplementary material for: Reproductive ecology and isolation of Psittacanthus calyculatus and P. auriculatus mistletoes (Loranthaceae)
Source: PeerJ. 2016 Sep 27;4:e2491. doi: 10.7717/peerj.2491 (PMC5045876; doi:10.7717/peerj.2491)
Supplement: Supplemental Information 1 — IDs reported below refer to accession numbers in the Instituto de Ecología, AC (XAL) herbarium. [file peerj-04-2491-s001.docx]

**Table S1 Voucher information of the *Psittacanthus* populations used in the study.** IDs reported below refer to accession numbers in the Instituto de Ecología, AC (XAL) herbarium.

| **Species** | **Location** | **Altitude**  **(m asl)** | **Latitude**  **(N)** | **Longitude**  **(W)** | **Voucher information** |
| --- | --- | --- | --- | --- | --- |
| *Psittacanthus calyculatus* | Jalisco, San José de Gracia | 2025 | 20°41´47´´ | 102°33´22´´ | XAL, E. Ruiz Sánchez 307 |
| *Psittacanthus calyculatus* | Jalisco, Gómez Farías | 1360 | 19°52´16´´ | 103°31´47´´ | BAJIO, A. González s/n |
| *Psittacanthus calyculatus* | Tlaxcala, Tlaxcala | 2200 | 19°17´00´´ | 98°14´00´´ | UAT, C. Lara s/n |
| *Psittacanthus calyculatus* | Tlaxcala, Nativitas and Tetlatlahuca | 2200 | 19°11´24´´ | 98°17´14´´ | XAL, A. Ortiz Rodríguez 751 |
| *Psittacanthus calyculatus* | Tlaxcala, San Luis Teolocholco | 2593 | 19°15´33´´ | 98°08´12´´ | XAL, M. J. Pérez-Crespo s/n |
| *Psittacanthus calyculatus* | Oaxaca, Santiago Matatlán | 1784 | 16°50´53´´ | 96°22´18´´ | XAL, A. Ortiz Rodríguez 750 |
| *Psittacanthus auriculatus* | Oaxaca, Santiago Matatlán | 1784 | 16°50´53´´ | 96°22´18´´ | XAL, A. Ortiz Rodríguez 749 |
| *Psittacanthus schiedeanus* | Oaxaca, Santiago Comaltepec | 848 | 17°41´23´´ | 96°20´13´´ | XAL, A. Ortiz-Rodríguez 754 |
| *Psittacanthus mayanus* | Yucatán, Hunucmá | 10 | 21º 02´ 58´´ | 89º 54´ 38´´ | – |
| *Psittacanthus mayanus* | Yucatán, Cuxtal | 11 | 20º 54´ 37´´ | 89º 37´ 15´´ | – |
| *Psittacanthus mayanus* | Chiapas, Ocozocuautla | 814 | 16º 47´ 47´´ | 93º 24´ 30´´ | XAL, Y. Licona Vera 017 |
